# Expression of G-protein inwardly rectifying potassium channels (GIRKs) in lung cancer cell lines

**DOI:** 10.1186/1471-2407-5-104

**Published:** 2005-08-18

**Authors:** Howard K Plummer, Madhu S Dhar, Maria Cekanova, Hildegard M Schuller

**Affiliations:** 1 Molecular Cancer Analysis Laboratory, Department of Pathobiology, College of Veterinary Medicine, University of Tennessee, Knoxville, TN 37996-4542, USA; 2 Molecular Cancer Analysis Laboratory, Department of Pathobiology, and Department of Large Animal Clinical Sciences, College of Veterinary Medicine, University of Tennessee, Knoxville, TN 37996-4542, USA; 3 Experimental Oncology Laboratory, Department of Pathobiology, College of Veterinary Medicine, University of Tennessee, Knoxville, TN 37996-4542, USA

## Abstract

**Background:**

Previous data from our laboratory has indicated that there is a functional link between the β-adrenergic receptor signaling pathway and the G-protein inwardly rectifying potassium channel (GIRK1) in human breast cancer cell lines. We wanted to determine if GIRK channels were expressed in lung cancers and if a similar link exists in lung cancer.

**Methods:**

GIRK1-4 expression and levels were determined by reverse transcription polymerase chain reaction (RT-PCR) and real-time PCR. GIRK protein levels were determined by western blots and cell proliferation was determined by a 5-bromo-2'-deoxyuridine (BrdU) assay.

**Results:**

GIRK1 mRNA was expressed in three of six small cell lung cancer (SCLC) cell lines, and either GIRK2, 3 or 4 mRNA expression was detected in all six SCLC cell lines. Treatment of NCI-H69 with β_2_-adrenergic antagonist ICI 118,551 (100 μM) daily for seven days led to slight decreases of GIRK1 mRNA expression levels. Treatment of NCI-H69 with the β-adrenergic agonist isoproterenol (10 μM) decreased growth rates in these cells. The GIRK inhibitor U50488H (2 μM) also inhibited proliferation, and this decrease was potentiated by isoproterenol. In the SCLC cell lines that demonstrated GIRK1 mRNA expression, we also saw GIRK1 protein expression. We feel these may be important regulatory pathways since no expression of mRNA of the GIRK channels (1 & 2) was found in hamster pulmonary neuroendocrine cells, a suggested cell of origin for SCLC, nor was GIRK1 or 2 expression found in human small airway epithelial cells. GIRK (1,2,3,4) mRNA expression was also seen in A549 adenocarcinoma and NCI-H727 carcinoid cell lines. GIRK1 mRNA expression was not found in tissue samples from adenocarcinoma or squamous cancer patients, nor was it found in NCI-H322 or NCI-H441 adenocarcinoma cell lines. GIRK (1,3,4) mRNA expression was seen in three squamous cell lines, GIRK2 was only expressed in one squamous cell line. However, GIRK1 protein expression was not seen in any non-SCLC cells.

**Conclusion:**

We feel that this data may indicate that stimulation of GIRK1 or GIRK2 channels may be important in lung cancer. Stimulation of GIRK channels and β-adrenergic signaling may activate similar signaling pathways in both SCLC and breast cancer, but lead to different results.

## Background

Recent studies in human cancer cell lines or in animal models have shown that the growth of adenocarcinomas of the lungs, pancreas and colon are under β-adrenergic control [1-5]. The tobacco carcinogenic nitrosamine 4-(methylnitrosamino)-1-(3-pyridyl)-1-butanone (NNK) has recently been identified as a high affinity β-adrenergic agonist that stimulated the growth of pulmonary and pancreatic adenocarcinomas in vitro and in animal models [1,3,5]. Expression of mRNA that encodes a G-protein coupled inwardly rectifying potassium channel (GIRK1) has been shown in tissue samples from approximately 40% of primary human breast cancers tested [6], and this expression of GIRK1 was associated with a more aggressive clinical behavior. Increases in GIRK currents by β-adrenergic stimulation have been reported in adult rat cardiomyocytes and in *Xenopus laevis *oocytes coexpressing β_2_-adrenergic receptors and GIRK1/GIRK4 subunits [7]. In addition, in rat atrial myocytes transiently transfected with β_1 _or β_2 _adrenergic receptors, the β-adrenergic agonist isoproterenol (Iso) stimulated GIRK currents, whereas this stimulation was not seen in non-transfected cells [8]. Previous data from our laboratory has indicated that there is a functional link between the β-adrenergic receptor pathway and the G-protein inwardly rectifying potassium channel (GIRK1) in breast cancer cell lines and these pathways were involved in growth regulation of these cells [9,10]. Additional data from our laboratory has also indicated that the normal breast epithelial cell line MCF 10A lacks GIRK1 expression [10].

Established risk factors for breast cancer include age, increased hormone exposure, alcohol consumption and family history as well as many other factors [11]. Smoking is a controversial risk factor for the development of this malignancy [12-14]. However, increases in pulmonary metastatic disease have been seen in smokers with breast cancer [15]. In addition, a study of 141,000 women showed a significantly increased risk of developing lung cancer for breast cancer patients, possibly due to interactions between radiotherapy and smoking [16]. An eight-fold increase in breast cancer risk has been seen in women who were smokeless tobacco users [17]. Smokeless tobacco has higher levels of nicotine and the tobacco carcinogen NNK than cigarette smoke [18]. However, many types of lung cancer are smoking related [19].

In order to investigate possible similarities between breast cancer and lung cancer, we wanted to determine if GIRK channels are expressed in human lung cancers and if β-adrenergic signaling is also involved in lung cancer. Voltage gated K^+^, Na^+ ^and Ca^2+ ^channels have been shown in the small cell lung cancer (SCLC) cell lines NCI-H128, NCI-H69 and NCI-H146 by measurement of currents [20]. These voltage activated K^+ ^channels in the SCLC cell lines have a role in modulating cell proliferation [21]. A classical inwardly rectifying potassium channel not linked with G-proteins has been shown in a subclone of NCI-H69, H69AR, a subline with overexpressed multidrug resistance associated protein, but these currents were not seen in the original H69 cell line [22]. Inwardly rectifying potassium currents have also been measured in RERF-LC-MA SCLC cells, and these cells express the Kir2.1 mRNA and protein [23]. GIRK1 expression has been shown in tissue specimens from patients with non-small lung cancer and this expression was associated with lymph node metastasis [24]. Prior to this study, GIRK channels have not been identified in lung cancer cell lines or SCLC cell lines and clinical samples. In the present research we have screened small cell and non-small cell lung cancer cell lines and several normal lung cell types for GIRK mRNA and protein expression.

## Methods

### Cell culture

The human SCLC cell lines NCI-H69 (H69), NCI-H146 (H146), NCI-H187 (H187), NCI-H209 (H209), and NCI-H526 (H526), the human adenocarcinoma cell lines NCI-H322 (H322), NCI-H441 (H441), and A549, the carcinoid cell line NCI-H727 (H727), and the squamous cell lines NCI-H226 (H226), NCI-H2170 (H2170), and NCI-H520 (H520) were purchased from the American Type Culture Collection (Manassas, VA). The human SCLC cell line WBA [25] was a gift of Dr. G. Krystal, Medical College of Virginia. All cancer cell lines except A549 were maintained in RPMI medium supplemented with fetal bovine serum (10% v/v), L-glutamine (2 mM), penicillin (100 U/ml) and streptomycin (100 μg/ml) at 37°C in an atmosphere of 5% CO_2_. A549 cells were grown in Hams F12 media with supplements as above. Human small airway epithelia cells (SAEC) were purchased from Clonetics/BioWhittaker (Walkersville, MD). These primary cells were maintained in SAEC basal medium with supplements (Clonetics) at 37°C in an atmosphere of 5% CO_2_. Fresh surgical tissue samples were collected from patients at the University of Tennessee (UT) Graduate School of Medicine's Cancer Center and processed for reverse transcription polymerase chain reaction (RT-PCR). The collection of tissue was approved by the UT Institutional Review Board, and the authors have been certified by the NIH Office of Human Subjects Research. Cultures of fetal hamster pulmonary neuroendocrine cells (PNEC) were established from fetal lung periphery harvested on day 15 of gestation as previously described [26,27]. Studies with fetal hamster cells were approved by the UT Institutional Animal Care and Use Committee. Exposure of cells to isoproterenol (Iso), the GIRK inhibitor U50488H (U5) [28] (Sigma, St. Louis, MO), or ICI 118,551 (ICI) (Tocris, Ballwin, MO) for experiments was as detailed in the figure legends or results.

### RT-PCR

RNA from cell cultures, adult hamster brain, PNEC and from fresh surgical tissue samples was isolated by guanidine isothiocyanate/cesium chloride ultracentrifugation [29] or by an Absolutely RNA kit (Stratagene, La Jolla, CA). RT-PCR was done as previously described [10]. The GIRK3 primers are forward 5'-gtgaccagcttcctccagac-3' and reverse 5'-gctaccatcttcccatccaa-3' which amplifies a 317 bp fragment (bases 1421–1737, Genbank Acession # NM_004983). PCR conditions are 94°C, 30 sec; 55°C, 30 sec; 72°C, 45 sec for 40 cycles. Cyclophilin primers were used an internal control (Ambion, Austin, TX).

### Real-time PCR

Real-time PCR was done as previously described [10]. GIRK1 primers-forward 5'-ctctcggacctcttcaccac-3' and reverse 5'-gccacggtgtaggtgagaat-3' (bases 398–477, Genbank Acession # NM002239) and the internal TaqMan probe is 6-FAM-tcaagtggcgctggaacctc-TAMRA (bases 429–449), annealing 62°. GIRK2 primers-forward 5'-gacctgccaagacacatcag-3' and reverse 5'-cggtcaggtagcgataggtc-3' (bases 766–886, Genbank Acession # U52153) and the internal TaqMan probe is 6-FAM-gtgcaatgttcatcacggcaac-TAMRA (bases 837–859), annealing 56°. GIRK4 primers-forward 5'-agcgctacatggagaagagc-3' and reverse 5'-aagttgaagcgccacttgag-3' (bases 241–358, Genbank Acession # L47208) and the internal TaqMan probe is 6-FAM-accggtacctgagtgacctcttca-TAMRA (bases 301–324), annealing 62°. Reactions were run on a Cepheid SmartCycler (Sunnyvale, CA). Reaction conditions are 200 μM dNTPs, 0.3 μM gene specific primers, 0.2 μM TaqMan probe, 4 mM (GIRK1) or 6 mM (GIRK2or4) magnesium acetate, 2 μl cDNA and 1.5 U MasterTaq (Eppendorf, Westbury, NY) and MasterTaq buffer in a final volume of 25 μl. In some experiments, 18S primers were used an internal control (Qiagen, Valencia, CA).

### Proliferation assay

Cells were counted and plated in RPMI without serum or phenol red at a density of 50,000 cells/well with appropriate treatments in a volume of 500 μl. Cells were allowed to grow for 48 hours, and proliferation was measured by a cell proliferation assay for 5-bromo-2'-deoxyuridine (BrdU) according to the manufacturer's instructions (Roche, Indianapolis, IN), N = 5 for each treatment group.

### Western blots

Cell pellets were collected and membrane protein was isolated with the ReadyPrep protein extraction kit (signal) (Biorad, Hercules, CA). Protein levels were determined using the RCDC kit (Biorad). Aliquots of 20–30 μg protein were boiled in 3× loading buffer (New England Biolabs, Beverly, MA) for two minutes, then loaded onto 12% Tris-glycine-polyacrylamide gels (Cambrex, Rockland, ME), and transferred electrophoretically to nitrocellulose membranes. Membranes were incubated with the primary antibody (GIRK1; Upstate Biotechnology, Lake Placid, NY). In all western blots, membranes were additionally probed with an antibody for actin (Sigma) to ensure equal loading of protein between samples. The membranes were then incubated with appropriate secondary antibodies (Rockland, Gilbertsville, PA or Molecular Probes, Eugene OR). The antibody-protein complexes were detected by the LiCor Odyssey infrared imaging system (Lincoln, NE).

## Results

**Figure 1:**
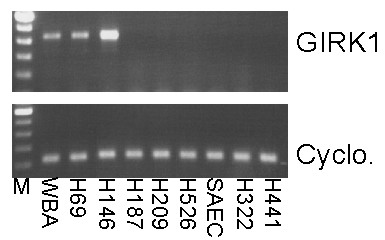
**Expression of GIRK1 in some SCLC cell lines but not in normal SAE cells, nor in adenocarcinoma Clara cell phenotype cell lines**. Top panel: GIRK1 is expressed in WBA, H69, and H146 SCLC cell lines but not in H187, H209 and H526 SCLC cell lines. GIRK1 is also not expressed in normal SAE cells, nor is it expressed in H322 or H441 adenocarcinoma Clara cell lines. Bottom panel: Cyclophilin, used as a positive reaction control was seen in all samples. For all gene expression experiments, negative control reactions were performed and found to be negative. The bands on the agarose gels were consistent with the expected sizes: GIRK1-441 bp; cyclophilin-216 bp; M-100 bp.

**Figure 2:**
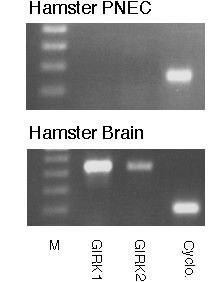
**Lack of GIRK1 and 2 expression in hamster PNEC cells**. Top panel: Pulmonary neuroendocrine cells were isolated from fetal hamsters on the 15^th ^day of gestation. Neither GIRK1 nor GIRK2 was expressed in normal PNEC. Bottom panel: Tissue was isolated from adult hamster brain to determine if the lack of expression in PNEC was not due to species differences in PCR primers. Both GIRK1 and GIRK2 were expressed in hamster brain. Cyclophilin, used as a positive reaction control was seen in the samples. For all gene expression experiments, negative control reactions were performed and found to be negative. The bands on the agarose gels were consistent with the expected sizes: GIRK1-441 bp; GIRK2-438 bp; cyclophilin-216 bp, M-100 bp.

In order to investigate possible expression similarities between breast cancer and lung cancer, we wanted to determine if GIRK channels are expressed in human lung cancers. Expression of mRNA for the GIRK1 channel was seen in three of six SCLC cell lines. GIRK 1 was expressed in the SCLC cell lines H69, H146 and WBA (Figure 1). Since GIRK1 cannot form channels alone, it must assemble with GIRK2, 3 or 4 [reviewed in [30]]. GIRK2 & 4 expression was determined in the six SCLC cell lines using real-time PCR. GIRK 4 expression was found in all six SCLC cell lines, and GIRK2 was found in all six SCLC cell lines with the exception of H146 and H187 (Table 1). In these experiments, real-time PCR was used as a second method of determining gene expression. Since gene expression was determined by only one sample (similar to gene expression studies by RT-PCR) no comparisons can be made between C_T _values for the samples. We wished to compare GIRK1 expression in SCLC to normal primary cells. GIRK1 was not expressed in the normal human SAE cells (Figure 1). Normal SAE cells express GIRK4 but do not express GIRK2 (data not shown). SCLC shares phenotypic and functional features with pulmonary neuroendocrine cells (PNEC), one of the possible origins of this cancer type [31]. Neither GIRK1 nor GIRK2 mRNA expression was found in normal tissue from PNEC cells isolated from prenatal hamster lungs using the primers designed for human GIRK1 and 2 (Figure 2). In order to determine if this result was due to species differences in GIRK1 and 2 between humans and hamsters, tissue was isolated from hamster brain. In both cases, mRNA from GIRK1 and 2 were found to be expressed in hamster brain (Figure 2). However, our GIRK4 primers were found not to work in hamster tissue (data not shown).

**Figure 3:**
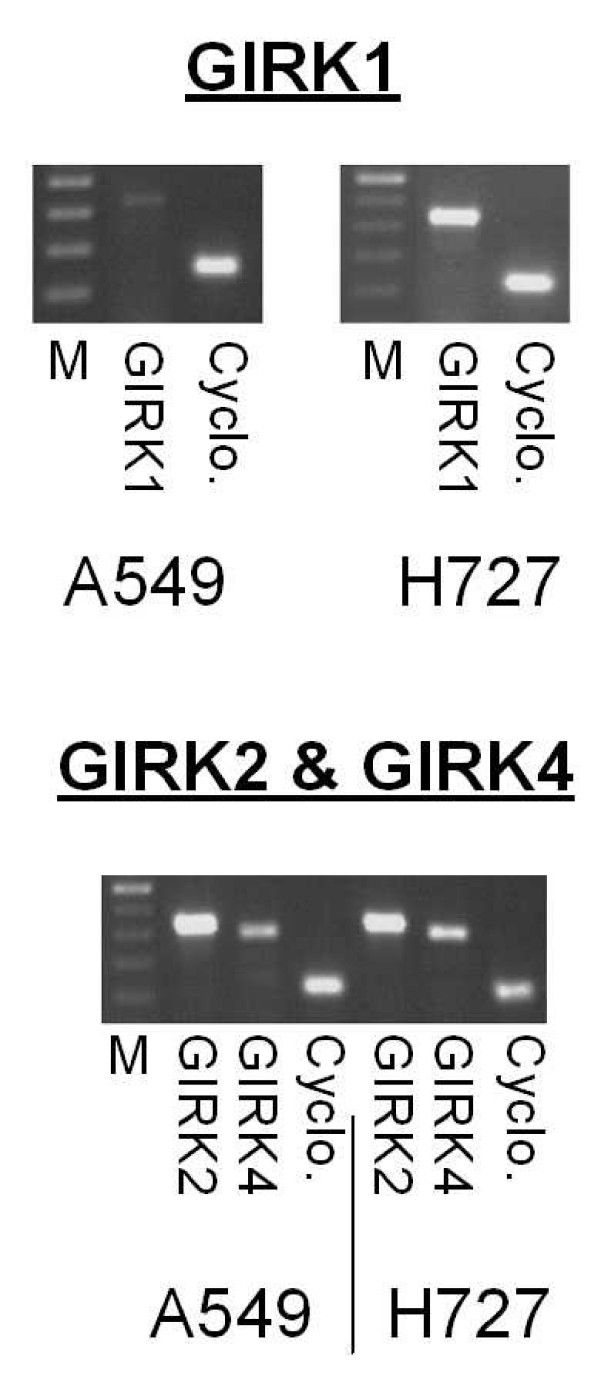
**Expression of GIRK channels in adenocarcinoma alveolar type II cell phenotype (A549) and carcinoid (H727) cell lines**. Top panel: GIRK1 was expressed in both A549 and H727 cell lines. Bottom panel: Both A549 and H727 expressed GIRK2 and GIRK4. Cyclophilin, used as a positive reaction control was seen in both samples. For all gene expression experiments, negative control reactions were performed and found to be negative. The bands on the agarose gels were consistent with the expected sizes: GIRK1-441 bp; GIRK2-438 bp; GIRK4-401 bp; cyclophilin-216 bp; M-100 bp.

**Figure 4:**
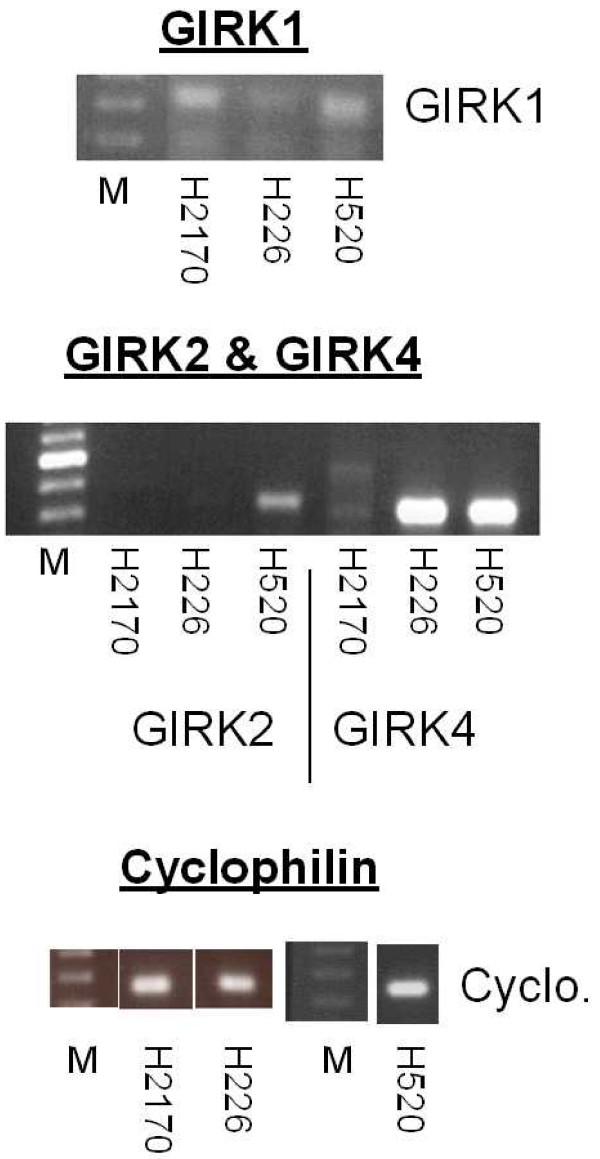
**Differences in GIRK expression in three squamous carcinoma cell lines**. Top panel: GIRK1 was expressed in all three squamous cell lines, H2170, H226, and H520. Middle panel: GIRK2 is only expressed in H520 cell line, but not in H2170 or H226. GIRK4 was expressed in all three squamous cell lines, H2170, H226, and H520. Bottom panel: Cyclophilin, used as a positive reaction control was seen in all samples. For all gene expression experiments, negative control reactions were performed and found to be negative. The bands on the agarose gels were consistent with the expected sizes: GIRK1-441 bp; GIRK2-438 bp; GIRK4-401 bp; cyclophilin-216 bp; M-100 bp.

We wanted to determine if GIRK channels were also expressed in human non-SCLC (NSCLC) cancers. GIRK1 was not expressed in two adenocarcinoma cell lines of Clara cell phenotype, H322 and H441 (Figure 1). However, three GIRK channels (1,2,4) were expressed in both the human adenocarcinoma cell line with alveolar type II cell phenotype (A549) and human carcinoid cell line expressing neuroendocrine features (H727) (Figure 3). GIRK1 and 4 are expressed in three squamous cell lines, H2170, H226, and H520 (Figure 4). GIRK2 is only expressed in the H520 cell line, but not in either H2170 or H226 cell lines (Figure 4).

**Figure 5:**
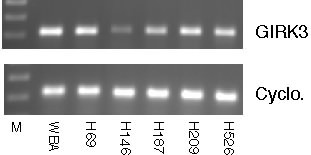
**Expression of GIRK3 in SCLC cell lines**. Top panel: GIRK3 expression was seen in all six SCLC cell lines, in WBA, H69, H146, H187, H209, and H526. Bottom panel: Cyclophilin, used as a positive reaction control was seen in all samples. For all gene expression experiments, negative control reactions were performed and found to be negative. The bands on the agarose gels were consistent with the expected sizes: GIRK3-317 bp; cyclophilin-216 bp; M-100 bp.

**Figure 6:**
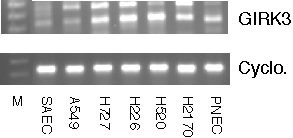
**GIRK3 expression in NSCLC cell lines and in normal cells**. Top panel: GIRK3 was expressed in normal human SAEC and hamster PNEC as well as being expressed in all NSCLC cell lines tested. These NSCLC cell lines are: A549, H727, H226, H520, and H2170. Bottom panel: Cyclophilin, used as a positive reaction control was seen in all samples. For all gene expression experiments, negative control reactions were performed and found to be negative. The bands on the agarose gels were consistent with the expected sizes: GIRK3-317 bp; cyclophilin-216 bp; M-100 bp.

**Figure 7:**
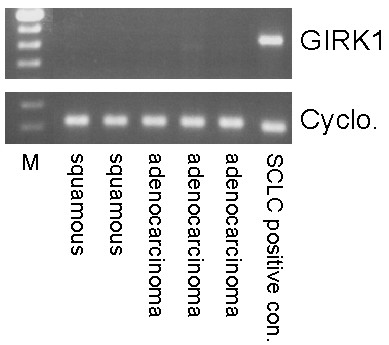
**Lack of GIRK1 expression in fresh surgical tissue samples of pulmonary adenocarcinomas or pulmonary squamous carcinomas**. Top panel: GIRK1 was not expressed in two pulmonary squamous carcinomas or three adenocarcinomas obtained from cancer patients. Bottom panel: Cyclophilin, used as a positive reaction control was seen in all samples. For all gene expression experiments, negative control reactions were performed and found to be negative. The bands on the agarose gels were consistent with the expected sizes: GIRK1-441 bp; cyclophilin-216 bp; M-100 bp.

Although the predominant GIRK heterotetramers seem to be GIRK1/2 and GIRK1/4 [reviewed in [30]], GIRK3 expression may also be important in lung cancer. GIRK3 expression in normal cells, SCLC cell lines and NSCLC cell lines was examined. GIRK3 expression was seen in all six SCLC cell lines (Figure 5). GIRK3 was expressed in normal cells (SAEC, PNEC) and in the five NSCLC cell lines (A549, H727, H2170, H226, H520) (Figure 6). A summary of all the above experiments indicating gene expression data for the cell lines and normal cells is shown in Table 2. We also wanted to determine if GIRK1 was expressed in fresh surgical tissue samples. A limited number of samples of pulmonary adenocarcinomas (three) or pulmonary squamous carcinomas (two) were collected from patients at the UT Graduate School of Medicine's Cancer Center. None of these tissue samples expressed GIRK1 (Figure 7).

**Figure 8:**
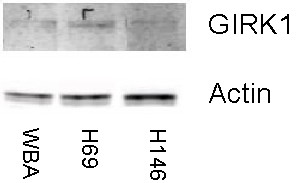
**GIRK1 protein expression in SCLC cells that express GIRK1 mRNA as assessed by western blot analysis**. In the three SCLC cell lines that expressed GIRK1 mRNA (Figure 1), membrane protein expression was determined. Top panel: All three SCLC cell lines (WBA, H69, H146) expressed GIRK1 membrane protein. Bottom panel: Actin was used as a control for equal sample loading. The bands are consistent with the expected size: GIRK1-62 kDa; actin-42 kDa.

For functional GIRK channels in lung cells, protein expression is needed as well as gene expression. We determined GIRK1 protein expression in cell lines that express GIRK1 mRNA. Isolating enriched membrane protein using a Biorad kit, we found GIRK1 protein expression in the three SCLC cell lines that expressed GIRK1 mRNA, H69, H146 and WBA (Figure 8). In addition, in the NSCLC cell lines that express GIRK1 mRNA, GIRK1 protein expression was determined. GIRK1 protein expression was not seen in A549, H727, H2170, H226, and H520 (data not shown).

**Figure 9:**
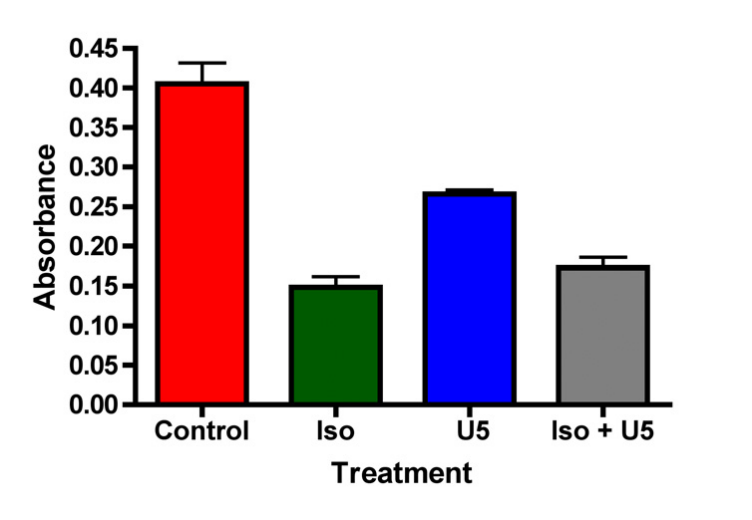
**Inhibition of proliferation in H69 cells by the β-adrenergic agonist isoproterenol and the GIRK channel inhibitor U50488H**. H69 cells were grown in the presence of isoproterenol or U50488H or a combination of Iso and U5 for 48 hours. Proliferation was determined by a BrdU assay. Both 10 μM Iso (p < 0.001) and 2 μM U5 (p < 0.001) inhibited proliferation with Iso having the greater effect. The effect of the GIRK inhibitor U5 on proliferation was potentiated by Iso (p < 0.001). N = 5.

Since β-adrenergic ligands affected both gene expression of GIRK1 and cell proliferation for breast cancer cell lines in our laboratory [10], we wanted to determine if β-adrenergic agonists and antagonists had effects on either gene expression or cell proliferation in SCLC cells. H69 cells were used for these experiments because it is one of the two cell lines that expressed mRNA for all four GIRK channels as well as GIRK1 protein. The second cell line, WBA, is not as well characterized in the literature. Exposure of MDA-MB-453 breast cancer cells for six days to the β-adrenergic antagonist propranolol increased the GIRK1 mRNA levels [10]. In the present experiments, we used two specific β-adrenergic antagonists to possibly induce changes in GIRK1 mRNA levels. The specific β_1 _antagonist atenolol (100 μM) had no effect on gene expression when H69 cells were treated for seven days (Table 3). However, when H69 cells were treated with 100 μM of the β_2 _adrenergic antagonist ICI daily for seven days, differences in gene expression were seen as determined by real-time quantitative PCR (Table 3). Calculating differences in gene expression using the 2^-ΔΔC^_T _Formula [32], ICI treatment caused a 1.47× decrease in GIRK1 gene expression p < 0.0003 (Table 3). H69 cells express mRNA for both β_1 _and β_2 _(data not shown). As indicated above, we wanted to determine if β-adrenergic ligands altered proliferation in H69 cells. We determined the effects of the β-adrenergic agonist Iso and the GIRK channel inhibitor U5 [28] on proliferation in H69 cells. Iso (10 μM) inhibited proliferation (p < 0.001) (Figure 9). The GIRK inhibitor U5 (2 μM) also inhibited proliferation (p < 0.001), and this decrease was potentiated by Iso (p < 0.001) (Figure 9).

## Discussion

This is the first report that describes the expression of mRNA for G-Protein Inwardly Rectifying Potassium Channels (GIRKs) in lung cancer cell lines. Previous reports have indicated that voltage gated K^+^, Na^+ ^and Ca^2+ ^channels have been shown in SCLC cell lines [20], and these voltage activated K^+ ^channels have a role in modulating cell proliferation [21]. Classical inwardly rectifying potassium channels not linked with G-proteins have been shown in a few SCLC cell lines [22,23]. Since GIRK1 cannot form functional channels by itself, other GIRK channels are needed [30]. All six SCLC cell lines tested express mRNA for either GIRK2 or GIRK4 indicating that functional GIRK potassium channels are possible in these SCLC cancer cell lines. GIRK1 has been shown in tissue samples from approximately 40% of primary human breast cancers tested [6], and this expression of GIRK1 was associated with a more aggressive clinical behavior. GIRK1 has also been shown to contribute to tumor progression in NSCLC human tumors [24].

Differences in GIRK channel mRNA expression was seen in different types of lung cancer. The three adenocarcinomas from the lung cancer patient samples did not express GIRK1, and a subset of lung adenocarcinoma cell lines (H322, H441) also did not express GIRK1. However, GIRK1 expression was seen in an adenocarcinoma cell line with alveolar type II cell phenotype (A549). These variations may be due to differences between the Clara cell and the alveolar type II lineage, or it may be due to the small sample size. The cell linage of the lung cancer patient samples is unknown. Data from our laboratory has also indicated differences in responses to anticancer agents between alveolar type II and Clara cells in the lungs [33]. Further research with additional cell lines and tissue samples is needed to determine if the differences in GIRK expression between alveolar type II cells and Clara cells is significant.

The two samples from squamous cancer patients also did not express GIRK1 mRNA. However, squamous carcinoma cells lines and a carcinoid cell lines do express GIRK1. In all NSCLC cell lines that express GIRK1, either GIRK2 or GIRK4 mRNA was expressed, indicating that functional GIRK potassium channels are possible in these non-SCLC cancer cell lines. Our data showing lack of GIRK1 expression in tumors from human adenocarcinomas and squamous cell carcinomas in is in contrast to Takanami et al. [24]. They found that in 72 NSCLC patients, 69% had high GIRK1 levels and 31% had low GIRK1 levels [24]. The differences may be due to our small sample size, or there may be other factors in cancer patients that affect GIRK1 expression. The expression of GIRK2 or GIRK4 has not been determined in previous studies.

The mRNA for GIRK1 & GIRK2 channels have not been detected in the normal cell lines tested, although the mRNA for GIRK4 channels was found in SAE normal cells. Our data indicates more differential expression of GIRK1 and GIRK2 than of GIRK4 in both SCLC and NSCLC cell lines. It is our hypothesis that GIRK4 is less important in lung cancer due to the fact it was expressed in all normal and cancerous cell lines. Further study is needed to determine the importance of GIRK1 and GIRK2 in lung cancer. In addition GIRK3 was found in all cell lines tested, including SAEC and normal hamster PNEC. Since GIRK3 expression was seen in all cells tested, we also feel it is unlikely to be a factor in tumor growth or progression. This hypothesis is supported by data indicating that one of the functions of GIRK3 is to inhibit plasma membrane expression of other GIRK subunits [34]. Further studies are needed to determine the role of GIRK3 in lung cancer.

Since protein expression would also be necessary for functional GIRK channels, we determined GIRK1 protein expression in cell lines that expressed GIRK1 mRNA. Expression of GIRK1 protein was seen in the three SCLC cell lines that express GIRK1 mRNA. This is the first report of GIRK1 protein expression in SCLC cell lines. We also determined GIRK1 protein expression in NSCLC cell lines. None of the NSCLC cell lines that expressed GIRK1 showed protein expression for GIRK1. We feel that this indicates that GIRK1 expression may be a larger factor in SCLC than in NSCLC. Further study is needed to elucidate these differences.

In the present study, we demonstrated a small decrease in GIRK1 mRNA expression (1.5×) in the H69 SCLC cell line after treatment for one week with the β_2 _adrenergic antagonist ICI daily for seven days. In contrast to this data, exposure of the breast cancer cell line MDA-MB453 for six days to the β-adrenergic antagonist propranolol (1 μM) increased the GIRK1 mRNA levels [10]. Treatment with the β_1 _antagonist, atenolol, for the same time period had no effect, indicating that the β_2_-adrenergic receptor is more important for changes in GIRK mRNA. GIRK currents have been shown to be increased in cells stimulated with the β-adrenergic agonist Iso in rat atrial myocytes transfected with β_1 _or β_2 _receptors [8]. Heterologous facilitation of GIRK currents by β-adrenergic stimulation was also seen in rat cardiomyocytes [7]. The differences in signaling pathways stimulated by β-adrenergic ligands in breast cancer and SCLC remain to be elucidated.

The tobacco carcinogen 4-(methylnitrosamino)-1-(3-pyridyl)-1-butanone (NNK), a high affinity agonist for β-adrenergic receptors, stimulated DNA synthesis in two breast cancer cell lines, an effect inhibited by ICI [9]. In the present report the β-adrenergic agonist, Iso, inhibited proliferation in H69 SCLC cells, also indicating opposite effects between breast cancer and small cell lung cancer with β-adrenergic agonists. The GIRK channel inhibitor U5 [28] also inhibited proliferation. It is our contention that the same receptors and channels (β-adrenergic, GIRK) are in both breast cancer and lung cancer, and stimulation of these pathways can lead to different results. This could be important in devising therapies in women breast cancer patients that are also smokers. Another study has indicated down-regulation of protein synthesis and mRNA expression of voltage-dependent and calcium-activated potassium channels bronchial and bronchiolar smooth muscle cells in rats by chronic smoking [35]. No differences were found in protein extracted from the lung, however.

Further studies are needed with RNA interference (RNAi) or small interfering RNA (siRNA) and additional β-adrenergic agonists and antagonists to further determine the effects of GIRK1 and GIRK2 on β-adrenergic signaling in human lung cancer, especially studies on differences in GIRK protein expression. However, it appears that β-adrenergic signaling and GIRK channels are important in both SCLC and breast cancer. These findings may be important in devising therapies based on β-adrenergic signaling and GIRK channel expression in lung cancers that express GIRK channels or β-adrenergic receptors.

## Conclusion

We feel that this data may indicate that stimulation of GIRK1 or GIRK2 channels may be important in lung cancer. Stimulation of GIRK channels and β-adrenergic signaling may activate similar signaling pathways in both SCLC and breast cancer, but lead to different results.

## Competing interests

The author(s) declare that they have no competing interests.

## Authors' contributions

HP carried out the majority of experiments, designed the study, and helped draft the manuscript. MD carried out the GIRK western blots. MD and MC were involved in proliferation studies. MC was also involved in RT-PCR. HS helped draft the manuscript.

## Pre-publication history

The pre-publication history for this paper can be accessed here:



## Figures and Tables

**Table 1:** Expression of GIRK2 or GIRK4 in small cell lung cancer cell lines. Expression of either GIRK2 or GIRK4 was determined by real time PCR. Threshold values (C_T_) values are listed below.

Cell Line	GIRK2	GIRK4
WBA	19.82	30.56
H69	23.54	32.18
H146	NF	40.54
H187	NF	31.60
H209	24.53	24.64
H526	27.55	23.21

NF – No C_T _value calculated due to lack of expression. Gene expression was determined by one sample. No comparisons can be made between C_T _values for the samples.

**Table 2:** Summary of GIRK mRNA expression in lung cells. This is a compilation of data shown in Figures 1–6 & Table 1. Some of the indicated data was not shown.

	GIRK1	GIRK2	GIRK3	GIRK4
Normal primary				
SAEC	NF	NF	E	E
PNEC	NF	NF	E	X
SCLC				
WBA	E	E	E	E
H69	E	E	E	E
H146	E	NF	E	E
H187	NF	NF	E	E
H209	NF	E	E	E
H526	NF	E	E	E
NSCLC				
H322	NF	ND	ND	ND
H441	NF	ND	ND	ND
H549	E	E	E	E
H727	E	E	E	E
H2170	E	NF	E	E
H226	E	NF	E	E
H520	E	E	E	E

E – mRNA expression

NF – mRNA expression not found

ND – mRNA expression not determined

X – human primer not compatible with hamster

**Table 3:** Changes in gene expression of GIRK1 after seven day treatment of H69 cells with the β_2 _adrenergic antagonist ICI 118, 551 (ICI) (100 μM) and lack of changes after seven day treatment with β_1 _adrenergic antagonist atenolol (AT) (100 μM).

	Control	ICI	AT
GIRK1	21.186 ± 0.13	22.040 ± 0.06^a^	21.238 ± 0.06
18S	12.028 ± 0.17	12.326 ± 0.03	12.090 ± 0.11

Changes in gene expression were determined by real time quantitative PCR, and are expressed as threshold values (C_T_), N = 5. ^a ^p < 0.0003. Threshold/C_T _cycle values will be higher for samples with less mRNA expression.
